# Was It Relapsing Fever?

**Published:** 1890-12

**Authors:** A. D. Barr

**Affiliations:** Calamine, Ark.


### WAS IT RELAPSING FEVER ?*

BY A. D. BARR, M. D., CALAMINE, ARK.

The following is the description of a continued form of fever that came under my observation in the latter part of 1889. At first I thought it to be malaria, and treated it accordingly; but I soon found that antiperiodics had no influence over the disease.

---

*Read before Mississippi Valley Medical Association, Louisville, Ky., October 10, 1890.

I then abandoned all treatment except to modify the symptoms when they were dangerous. When the fever exceeded 103° F., an antipyretic was given, and this was always acetanilide or quinine. When the nausea and vomiting were prominent some form of opium was given.

During the first intervals nux vomica, arsenic and quinine were administered to prevent a return of the paroxysms, but without success. The natural course of the disease was carefully studied, uninfluenced by treatment, and the symptoms noted at the time, and furnish the data of this article. It was a self-limited, continued form of fever, the distinctive feature of which, was three or more relapses. The recurrence of the fever, as before stated, was the distinctive feature. Abruptness of invasion was characteristic of the disease.

There was no prodromic or incubative stage. The seizures were never ushered in by a distinct chill, though chilly sensations were complained of, followed by a rise of temperature, usually sufficient to cause the patient to take to bed at once, though it sometimes required one, two or even three days for the temperature to be become sufficiently elevated to produce such a result. Perspiration seldom occurred until the fever began to decline. The fever attained quickly to considerable intensity, often reaching 103° or 104° F., within twenty-four hours. In one case the fever reached 104½° F., on the evening of the first day; this however was a relapse, instead of the primary attack. During the paroxysms the temperature denoted a persistent intensity of fever, ranging from 100° to 105° F. There were no oscillations; the temperature either gradually or rapidly reached its maximum, and then maintained about the same elevation for from one to four days, and sometimes longer, and usually subsided gradually. The duration of the primary attack varied from six to twelve days.

During the intermission, the absence of fever and all other symptoms was complete. The period of intermission varied from two to ten days ; the average was about seven. The relapse, like the primary attack, was sudden, and was also generally ushered in by chilly sensations ; though the first symptom was sometimes an elevation of temperature. The fever in the relapse suddenly became more or less elevated ; usually more elevated than in the primary.

The relapses also generally subsided gradually, and with moderate perspiration. The duration of the relapses was from four to ten days; and the greatest number of relapses was five. Of symptoms referable to the digestive system, nausea and vomiting occurred often enough to be somewhat characteristic. They were generally prominent features, but they were not always present. They were frequently so severe that the patient was in danger of sinking from exhaustion or collapse. The vomited matter generally contained bile, and not infrequently blood. The tongue was usually coated with a white fur, but sometimes became dry and cracked. Diarrhœa was very infrequent. Constipation was the rule. Tenderness over the epigastric and iliac regions occurred to a very moderate degree. The appetite was often impaired, but not to the extent that is usual in most diseases where the temperature is elevated to as high a degree as it was in this. Sometimes there was a notable craving for food, so much so that it was highly distinctive. During the paroxysms pains in the muscles of small of the back and the calves of the legs were complaned of. They were never entirely wanting, though they varied in intensity. The perception was not blunted in this as in various other diseases. Delirium was present sometimes, but when it was, it was of that character that is likely to occur when there is a high febrile movement without regard to the existing malady. The face was usually flushed. The amount of urine was usually decreased. The amount of decrease in one instance was complete suppression for twenty-four hours, and the kidneys acted badly for several days thereafter, which in turn was followed by an excessive diuresis. A copious flow was apt to occur about the cessation of each seizure.

Regarding the cause of the disease, I am unable to give any satisfactory answer. In one case it seemed probable that it was contracted by contagion, as the child was in frequent contact with a young lady suffering with the same disease. The disease seemed to be developed spontaneously. In regard to treatment, all that I could do was to modify the symptoms. The disease was essentially a self-limited one, and was uninfluenced by all treatment instituted by me. Quinine given in five to ten grain doses, from two to six hours apart seemed to aggravate by increasing the nausea and vomiting. Calomel seemed to retard convalescence. The nausea and vomiting was best controlled by morphine and listerine. In the first stage, pains were often so severe as to call for an anodyne, but as a rule, upon reduction of the temperature below 100° F., they subsided until they again became intense enough to call for a repetition of the antipyretic. Good, plain, wholesome food was allowed.

